# Supplementary material for: Evaluation and optimization of outdoor wind environment in block based on space syntax and CFD simulation
Source: PLoS One. 2024 Mar 28;19(3):e0297683. doi: 10.1371/journal.pone.0297683 (PMC10977889; doi:10.1371/journal.pone.0297683)
Supplement: Attachment — Submitted filename: Response to Reviewers.docx [file pone.0297683.s001.docx]

1) What does it mean when you say, ‘To optimize the wind environment of the block more accurately, it analyzed, and 8 evaluated the accessibility of walking space of the block by relying on space syntax.’ Could you please clarify or explain how the space syntax analysis could provide accurate results for the wind simulation?

**Response:** As the outdoor space of the block is too large, the wind environment optimization cannot take into account every space, and the low accessibility space with less pedestrian reach frequency will bring less benefit to the optimization. Therefore, this paper mainly focuses on the pedestrian wind environment. In order to maximize the quality of the pedestrian wind environment of the pedestrian space, the paper analyzes the pedestrian accessibility of the pedestrian space based on space syntax. The highly accessible Spaces with the highest pedestrian travel frequency are delineated, and the shape characteristics and wind environment of these Spaces are accurately analyzed and optimized to ensure the effectiveness of wind environment optimization.

2) Number of references needs to be increased. It requires more work on reviewed papers regarding pedestrian wind comfort and urban airflow simulation.

**Response**: In the introduction part, the paper increases the number of citations of research achievements in the field of pedestrian wind environment simulation, and summarizes them, which provides the basis for the research method of the paper.

3) The authors must provide detailed information on input data and boundary condition setting in the CFD model.

**Response**: The authors add detailed information about the input data of the model and the setting of boundary conditions during the CFD simulation. The result is shown in Figures 6 and 7.

4) Even though the study calculated the residual errors of the CFD modeling, it is necessary to present validation results between the CFD modeling and field experiment.

**Response**: In order to ensure the rationality and feasibility of the simulation software and the selected simulation process, a comparison chart between the average field measured value and the simulated value has been added in the "Verification of simulation results" section, and specific data has been provided. Finally, it can be seen from the comparison chart that the curve changes have a relatively good agreement. It can reflect the change trend of actual wind speed, so it can prove the rationality and feasibility of the selection of simulation software and simulation process. The result is shown in Figure 9.

5) More information about the site selection and site characteristic are required. Do you have any site selection criteria?

**Response**: Lanzhou is located in a cold region in northwest China. Due to its geographical and climatic characteristics, urban blocks are usually enclosed. However, with the development of urbanization, the summer heat island effect of Lanzhou is constantly enhanced. The wind environment of enclosed blocks needs to be improved urgently. Therefore, the site selection standard of this study is that the block layout is enclosed and the flow of people is large, which is convenient to divide the high-accessibility area.

6) What research gap did this study observe from previous works? How do the study’s findings provide a significant contribution to the readers? Or any new insights will be provided in this study?

**Response**: The study and optimization of wind environment usually do not take into account the real needs of pedestrians. Some Spaces with good wind environment but few people can't really serve people. In this paper, space syntax is used to delineate highly accessible space areas, and the correlation analysis and improvement of wind environment and architectural form of these Spaces are carried out. In addition, the combination of space syntax and numerical simulation technology is also an innovation in the research methods and ideas of urban wind environment.

7) Please clarify why the study used space syntax and the airflow simulation model. It is necessary to present more fundamental concepts and numerical methods of the space syntax software. The parameter setting in space syntax should also be presented in this paper.

**Response**: The reasons for studying the use of space syntax and air flow simulation models are mentioned in the introduction. A large number of previous research results have proved that space syntax is a theory and method to study the relationship between spatial organization and human society through the quantitative description of human settlement spatial structure including buildings, settlements, cities and even landscapes, and has a better measurement of spatial accessibility. In addition, PHOENICS software and air flow simulation model are applicable to block scale. It can accurately reflect the wind environment at pedestrian height.
