# Supplementary material for: Evaluation and optimization of outdoor wind environment in block based on space syntax and CFD simulation
Source: PLoS One. 2024 Mar 28;19(3):e0297683. doi: 10.1371/journal.pone.0297683 (PMC10977889; doi:10.1371/journal.pone.0297683)
Supplement: Attachment — Submitted filename: Response to Reviewers.docx [file pone.0297683.s002.docx]

1.Novelty: The authors have mentioned optimization of the block in the abstract. However, the section discussing on optimization analysis does not perform any of the commonly used statistical methods used for wind analysis in the built environment. Looking at the qualitative patterns of the wind profile does not fit the concept of optimization analysis.
**Response:** According to the suggestions of experts and scholars, Photoshop is used to process the air age cloud image in the section of wind environment optimization analysis. That is, the air age value and area represented by different color blocks are calculated, and the weighted average method is used to calculate the average air age of the study area. Combined with the practical application of this paper, the intermediate wind speed ratio is used as the relative comfort zone wind speed ratio R_S_ to judge the area size that meets the comfort level of outdoor pedestrians at a height of 1.5m.

2. ABL Homogeneity: The residual from the software is shown to demonstrate ABL homogeneity analysis. This is an inaccurate method. The authors need to follow the standard methodology used in the literature to perform homogeneity analysis by performing the fetch test and measuring the wind and turbulence profile at the inlet and outlet.
**Response:** According to the suggestion, the horizontal homogeneity test of flow field is studied.

The atmospheric boundary layer should maintain horizontal homogeneity between upstream and downstream, so the inflow characteristics of computational fluid dynamics (CFD) simulations should be stable throughout the computational domain. In this study, the CFD numerical simulation of the wind tunnel test flow was carried out in a blank computational domain, and the wind speed flow characteristics at the center were extracted and compared with the inflow characteristics. As shown in Figures 5 , the CFD model established in this study basically meets the requirements of horizontal homogeneity. The wind speed characteristic curves of the inlet, center, and outlet are basically consistent.

3. Grid Refinement Studies: Authors have not performed any grid refinement studies to show the sensitivity of the results to the grid resolution.

**Response:** In the chapter of computational grid division, the grid form and precision used in the simulation of block wind environment are expounded. The specific operations are shown in Figure 4.

Other Issues:
There are a lot of figures included in the paper which can be dropped.
1. Figure 3 and 4 not required as there is no discussion of temperature.

**Response:** On the recommendation of the review experts, this image has been removed.
2. Table 1 is not required as it is available in the literature

**Response:** On the recommendation of the review experts, the table has been removed.
3. Figure 7 and 8 has no relevance to the paper. Can be removed.

**Response:** On the recommendation of the review experts, this image has been removed.
4. Figure 10 is not essential.

**Response:** On the recommendation of the review experts, this image has been removed.
5. Figure 11 should be removed and replaced with proper ABL homogeneity study.

**Response:** On the recommendation of the review experts, this image has been removed.

6. There is no Figure 14 and jumps to Figure 15

**Response:** The content has been revised according to the recommendations of the review experts.
7. Figures 17-19 are not required as it is common-sense knowledge
**Response:** On the recommendation of the review experts, this image has been removed.
8. Authors need to improve the resolution of the contour plots.

**Response:** The content has been revised according to the recommendations of the review experts.
